# Dietary Intake and Beliefs of Pregnant Women with Gestational Diabetes in Cape Town, South Africa

**DOI:** 10.3390/nu10091183

**Published:** 2018-08-28

**Authors:** Stephanie M. Krige, Sharmilah Booley, Naomi S. Levitt, Tawanda Chivese, Katherine Murphy, Janetta Harbron

**Affiliations:** 1 Division of Human Nutrition, Department of Human Biology, Faculty of Health Sciences, University of Cape Town, Cape Town 7925, South Africa; stephkrige@hotmail.com (S.M.K.); Sharmilah.booley@uct.ac.za (S.B.); 2 Division of Diabetes and Endocrinology, Department of Medicine, Faculty of Health Sciences, University of Cape Town, Cape Town 7925, South Africa; naomi.levitt@uct.ac.za; 3 Chronic Disease Initiative for Africa, Department of Medicine, Faculty of Health Sciences, University of Cape Town, Cape Town 7925, South Africa; tchivese@gmail.com (T.C.); katherine.murphy@uct.ac.za (K.M.); 4 MRC/Wits Developmental Pathways for Health Research Unit, Department of Paediatrics, Faculty of Medicine and Health Sciences, University of the Witwatersrand, Johannesburg 2000, South Africa; 5 Biostats Unit, Division of Epidemiology and Biostatistics, Faculty of Medicine and Health Sciences, Stellenbosch University, Cape Town 7599, South Africa

**Keywords:** hyperglycaemia first detected in pregnancy, gestational diabetes, GDM, dietary intake, beliefs, SSBs, fruits and vegetables, sugar intake, pregnancy nutrition

## Abstract

This study investigated the dietary intake of pregnant women with gestational diabetes mellitus (GDM) and their beliefs relating to the consumption of fruits and vegetables (F&V) and sugary foods and drinks. A cross-sectional study was conducted on 239 pregnant women with GDM in Cape Town. Dietary intake was assessed using a quantified Food Frequency Questionnaire and beliefs relating to food choices were assessed using the Theory of Planned Behaviour (TPB). The mean energy intake was 7268 KJ, carbohydrate was 220 (±104.5) g, protein 60.3 (±27.5) g and fat 67.7 (±44.2) g. The macronutrient distribution was 55% carbohydrates, 14.5% protein and 30.5% fat of total energy. The majority of the sample had inadequate intakes of vitamin D (87.4%), folate (96.5%) and iron (91.3%). The median (IQR) amount of added table sugar and sugar sweetened beverages (SSBs) was 4.0 (0.00–12.5) g and 17.9 (0.0–132.8) mL per day, respectively. Only 31.4% met the recommendation (400 g per day) for F&V. Beliefs that it was not easy to exclude sugary foods/drinks and that knowing how to control cravings for sugary foods/drinks are areas to target messages on the sugar content of SSBs. In conclusion, the dietary intake of these women was not optimal and fell short of several nutritional guidelines for pregnant women with hyperglycaemia. The strongly held beliefs regarding sugary foods/drinks may contribute to poor adherence to nutritional guidelines among pregnant women with GDM in South Africa.

## 1. Introduction

Pregnancies complicated by hyperglycaemia are classified as pre-existing diabetes or hyperglycaemia first detected in pregnancy, which includes both gestational diabetes mellitus (GDM) and overt diabetes [1,2]. The prevalence of hyperglycaemia in pregnancy has been increasing worldwide. The estimated global prevalence is 16.2%, with the vast majority being due to GDM diagnosed in women living in low and middle-income countries [3]. Mwanri et al. [4] reported an increase in the prevalence of hyperglycaemia first detected in pregnancy in sub-Saharan Africa over the last 50 years. Recently, the prevalence of GDM in South Africa was estimated to be as high as 25.6% using the International Association of the Diabetes and Pregnancy Study Groups (IADPSG) diagnostic criteria [5]. The rise in number of women with hyperglycaemia in pregnancy is likely to be due to changes in lifestyle associated with urbanisation, including a Western style diet and sedentary lifestyle, which lead to overweight and obesity [6].

While there is no international consensus over the diagnostic criteria for GDM, it is well established that uncontrolled diabetes during pregnancy poses numerous risks for the mother and foetus [1,5]. The Hyperglycaemia and Adverse Pregnancy Outcome (HAPO) study [7] found that, outside of overt diabetes, there was an association between increasing blood glucose levels and a number of adverse pregnancy and birth outcomes such as birth weight above the 90th percentile, shoulder dystocia, neonatal hypoglycaemia, hyperbilirubinemia, preeclampsia and caesarean delivery. Although GDM usually resolves after child birth [8], it is associated with long-term health risks to the mother including postnatal depression, weight retention [9], GDM in future pregnancies and Type 2 Diabetes Mellitus in later life [10,11,12,13]. The consequences for the infant place them at risk of adiposity, impaired glucose tolerance and cardiovascular health problems in adulthood [9].

Optimal control of glycaemia is the key focus of treatment for women with GDM. Currently, guidelines for pregnant women with diabetes recommend initial diet and lifestyle intervention followed by oral hypoglycaemic agents and insulin, if diet alone does not achieve glycaemic targets [1,14,15]. After pregnancy, continuation of these healthy lifestyle practices is recommended for weight loss and reduced long-term health risks. While different associations have proposed dietary guidelines for women with hyperglycaemia in pregnancy, there is a lack of consensus on the recommended macronutrient distribution as summarized in Table 1.

Recent Cochrane reviews reported that dietary interventions have proven successful in: reducing the incidence of GDM and marginally lower fasting blood glucose levels at 32 to 36 weeks in pregnant women [9], meeting postpartum weight goals, decreasing postpartum depression and reducing the incidence of large-for-gestational age (LGA) and neonatal fat mass in pregnant women with GDM [16]. These dietary interventions have included a focus on macronutrient distribution [17], the promotion of a Mediterranean diet [18], a low glycaemic index (GI) diet [19] and increasing dietary fibre [20].

A few studies have investigated the diet of pregnant South African women [21,22,23,24,25,26,27,28,29]. These studies have reported high percentage of women with inadequate dietary intakes of key micronutrients such as iron, folate, vitamin A, vitamin C, calcium and zinc [22,23,26,28,29] as well as low intakes of fruits and vegetables (F&V) [26,28]. No studies to date could be traced that have investigated the diet of pregnant women with diabetes in Africa or South Africa. It is thus unknown whether they meet dietary guidelines and goals as proposed by various organizations for a healthy pregnancy and for optimal glucose control. In addition, investigating the underlying beliefs that shape dietary intake behaviours is needed to plan effective nutrition education programmes that promote lifestyle changes [30]. The primary aim of the current study was to investigate the dietary intake of pregnant women with GDM in Cape Town, South Africa and whether they adhere to established dietary recommendations in order to determine the dietary inputs needed in this population. The secondary aims of the study sought to investigate their beliefs related to sugary foods and drinks and F&V intake and the association between sociodemographic factors and dietary intake.

## 2. Materials and Methods

### 2.1. Study Design and Participants

A cross-sectional study design with an analytical component was used. The target population was pregnant women with hyperglycaemia first diagnosed in pregnancy attending Groote Schuur Hospital (GSH) or Mowbray Maternity Hospital (MMH) in Cape Town, South Africa. GSH is a tertiary referral hospital for high risk pregnancies while MMH is a secondary hospital for lower risk pregnancies. Participants were included if they were in the third trimester, thus ≥28 weeks’ gestational age and were screened for hyperglycaemia between 24–28 weeks gestational age and diagnosed by the hospital’s medical doctors with hyperglycaemia for the first time during the current pregnancy.

Many international organisations have adopted the IADPSG guidelines [35] as described by the World Health Organisation (WHO) for the diagnosis of hyperglycaemia first detected in pregnancy as GDM, when fasting glucose is ≥5.1–6.9 mmol/L, 1 h ≥ 10.0 mmol/L or 2 h ≥ 8.5–11.0 mmol/L or overt diabetes when fasting glucose ≥ 7.0 mmol/L or 2 h ≥ 11.1 mmol/L [2,36]. Both GDM and overt diabetes are first recognized during pregnancy, however in overt diabetes the diagnostic criteria for diabetes in non-pregnant adults are met, while GDM is a lesser degree of hyperglycaemia that is not clearly overt diabetes and usually resolves after pregnancy [1,2]. Although SEMDSA [1] also recommends the use of the aforementioned criteria, the diagnostic criteria currently used at facility level in South Africa are not consistent and decided independently by each facility. In the Western Cape public health sector a different diagnostic criteria are used [5]. The diagnostic criteria used at GSH and MMH and therefore the inclusion criteria for the study were as follows: impaired glucose tolerance (IGT) (fasting blood glucose of 5.6–6.9 mmol/L and/or 2 h OGTT between 7.8–11.0 mmol/L) or GDM (fasting blood glucose ≥ 7.0 mmol/L and/or OGTT ≥ 11.1 mmol/L) and in line with the National Institute for Health and Care Excellence (NICE) guidelines for the diagnosis of GDM if fasting blood glucose is ≥5.6 mmol/L and/or 2 h OGTT ≥ 7.8 mmol/L [37]. As the term IGT is not used in the diagnostic criteria for pregnant women according to IADPSG, WHO, NICE and other associations and all women in our study would be classified as GDM according to the NICE diagnostic criteria we refer to the study participants as women with GDM.

Women were excluded if they were younger than 18 years, had a multiple pregnancy or were diagnosed with T1DM or T2DM before the onset of this pregnancy. Treatment received in the form of pharmacotherapy, dietary and exercise guidelines or any other were not an exclusion criterion as it was difficult to establish the amount and type of exposure received in this population. The dietary and exercise recommendations that women with hyperglycaemia in pregnancy receive in the public health sector of South Africa are not standardized across facilities and provinces. In Cape Town patients could be referred to the dietitian on diagnosis of hyperglycaemia in pregnancy, however no standardized criteria for referrals exists. Patients are either seen on the day of diagnoses or scheduled an appointment in 1–2 weeks as an outpatient depending on the availability of the dietitian. An initial dietetic consultation is about 30 min and usually involves taking a diet history and providing recommendations to improve current dietary intake according to SEMDSA and the latest South African Food Based Dietary Guidelines [1,38]. Energy requirements, personalized meal plans, menus and diet exchanges are not calculated. The limited time for consultations and dietary inputs are due to the high number of patients requiring dietetic consultations together with the small number of dietitians employed in the public health sector. By 28 weeks, patients may or may not have seen a dietitian, it is not standard practice. Patients could also have been exposed to group nutrition talks as inpatients or as an outpatient, in the waiting room by a midwife on healthy eating during gestational diabetes pregnancy. Exercise recommendations are mostly not provided but in interspersed instances could be given if a biokineticist, physiotherapist or student interns are at the health care facilities.

### 2.2. Sample Size and Recruitment

According to Mostert et al. [28], in 2005, 23.9 to 26.1% of South African pregnant women had inadequate intakes of various macronutrients. Using this proportion (26.1%), a 99% confidence interval and 7.5% level of significance from an unlimited population size, a sample size of 228 was calculated using OpenEpi.

A sample of *n* = 239 pregnant women with GDM were recruited from antenatal clinics at GSH and MMH as well as in-patients at GSH using a consecutive sampling technique. The files of all patients attending the diabetic antennal clinic and the files of patients admitted in the ward were screened by fieldworkers. Patients that fitted the inclusion criteria, were provided with information on the study both verbally and by means of a written information sheet and were invited to participate in the study. At MMH and GSH, a private room was allocated for interviews to take place. Inpatients were interviewed at their bedside.

The study was approved by the University of Cape Town, Faculty of Health Sciences, Human Research Ethics Committee (HREC REF 229/2015 and 230/2015), participation was voluntary and each participant signed an informed consent form.

### 2.3. Questionnaire Development

An interview-administered questionnaire was developed for this study. The different sections were developed and reviewed by an expert panel of four dietitians to confirm the appropriateness of questions, coverage of core concepts and the level and comprehensibility of the questions. This ensured construct and content validity. A draft questionnaire was developed and reviewed several times before its finalization. It was then pilot tested on five people from the relevant population to check whether there was any difficulty in answering the questions and revised accordingly. All field workers were trained and standardised in the administration of the questionnaire. The completion of one questionnaire took forty to fifty minutes.

#### 2.3.1. Demographics and Disease Related History

Sociodemographic and obstetric history data included age, race, gestational age, number of pregnancies, previous GDM and number of living children. Self-rated questions on current physical activity level and food choices were reported as well as interest in being part of a wellness program for GDM.

#### 2.3.2. Socioeconomic Status

Socioeconomic status was assessed using the Living Standard Measurement (LSM) which was developed by the South African Advertising Research Foundation (SAARF) [39]. The LSM is a wealth measure segmentation tool to profile the South African consumer market and has been used in many different studies [40,41]. The questionnaire includes a list of 29 household items and respondents select all items they own. Each item on the LSM list has a weightage score. From the combination of household items in ownership by a participant, a LSM score is calculated using the LSM calculator [39]. Ten wealth groups have been identified based on the participants’ socioeconomic status from the lowest (LSM 1) to the highest (LSM 10) [42]. The LSM gives an indication of the food cash expenditure of the participant which can be as high as 70% of their total cash expenditure, in LSM categories 1 to 3, to as low as 5% of total cash expenditure in the wealthiest consumers [40].

#### 2.3.3. Dietary Intake Assessment

For the purpose of this study, a picture-sort [43] quantified Food Frequency Questionnaire (qFFQ) was developed to assess the usual dietary intake of the study participants after their diagnosis of GDM. The food list was compiled by an expert panel of registered dietitians using existing qFFQs that were used to assess dietary intake of educators from low socio-economic areas in the Western Cape [44] and pregnant women in Soweto, Johannesburg, [45] as well as the FFQ proposed in the Dietary Assessment and Education Kit (DAEK) [46] which was developed from extensive research to enable researchers to have a resource that was adapted to the local South African diet and available foodstuff. The FFQ included 103 food items, with some items having sub-item categories (see Supplementary file). In order to increase respondent accuracy in recalling foods consumed during the administration of the questionnaire, each food item on the FFQ list was represented by the appropriate visual card developed for the DAEK by Steyn and Senekal [46]. Study participants sorted the picture cards into 2 stacks according to foods they did and those they did not consume within the last two weeks. Using the cards from the stack of food items consumed over the last two weeks, the respondents were asked, to recall their portion size and their frequency of intake over the last two weeks. A small booklet derived from the DAEK manual, representing different portion sizes, was used to assist with portion sizes estimation. For data analyses, the household portion was converted into grams using the DAEK and then multiplied by the frequency of intake in the last two weeks and converted to daily intake. Each food item consumed on the FFQ was coded and the daily intake of energy, macro- and micronutrients were calculated for each participant using the South African food composition tables [47]. Participants who had an implausible daily energy intake of <2092 KJ (500 kcal) or >20,920 KJ (5000 kcal) [48] were excluded from data analyses (*n* = 9). In order to determine the adequacy of dietary intake the daily intake of protein (in grams), carbohydrates (in grams), fibre (in grams), vitamins and minerals was compared to the DRIs for pregnant women as established by the Institute of Medicine [34]. Proteins, carbohydrates and fats were computed as a percentage of total energy (TE) intake and categorized to reflect the percentage of women in our sample that consumed according to the macronutrient distribution recommendations of different international guidelines as summarized in Table 1. For instance, fat was categorized as % participants that consumed <30%, 30–34.9%, 35–40% and >40% of TE; protein was categorized as <10%, 10–15%, 15.01–20% and >20% of TE while carbohydrates were categorized as <40%, 40–44.9%, 45–50% and >50% of TE.

The daily intake of teaspoons of sugar was calculated for each participant by adding the total number of level teaspoons of sugar added to tea/coffee and used in porridge or vegetables. The daily intake of SSBs were calculated by adding the intake of squashes, fruit juice, carbonated beverages and sweetened milk drinks in millilitres. F&V intake was calculated by summing the grams of all vegetables (excluding potato which is high in carbohydrates) and all fruits (excluding avocado pear which is high in fat) eaten per day and were compared to the WHO recommendation of 400 g per day [21]. SSBs were compared to 0 mL as recommended by SEMDSA [1] and added table sugar to 0 g based on recommendation by WHO for total sugars added to foods to be <5% total energy [49]. This 5% could account for sugar added in food products such as tomato sauce, cereals and canned foods, thus recommending no added table sugar.

#### 2.3.4. Beliefs

The theory of planned behaviour (TPB) was used in order to understand what motivates behaviour change so as to help women with GDM adopt a healthier lifestyle. The TPB suggests that intention is the immediate precursor of behaviour [50]. Further the TPB states that intention is predicted by an individual’s attitude, subjective norms (the perceived social pressure to perform or not perform the behaviour) and perceived behavioural control (the perception of ease or difficulty of the particular behaviour), while each of these predictor constructs are determined or underlined by behavioural beliefs (about the consequences of performing the specific behaviour), normative beliefs (about the support/no support of specific referents of performing the specific behaviour) and control beliefs (about barriers or facilitators of the performing the specific behaviour), respectively. These beliefs are unique to each behaviour and target population [51]. It provides in-depth understanding of the behaviour within the specific population and context. To change an individual’s intention and behaviour with regards to a specific behaviour, these elicited beliefs need to be addressed/or challenged in intervention (or communication).

To develop the belief statements for inclusion in our study questionnaire we followed the TPB questionnaire development guidelines outlined by Francis et al. [52]. Hence, a formative qualitative study using in depth interviews with 50 women with GDM at MMH and GSH was conducted (results not reported in this article). This was done to elicit the salient behavioural, normative and control beliefs (most commonly held beliefs that is, those that first come to mind), in relation to specific dietary behaviours (sugary foods and drinks and F&V) of the target population. The most frequent salient beliefs were identified using a process recommended by Krueger and Casey [53] for managing qualitative data and converted into incomplete sentences with bipolar endpoints (agree vs. disagree) for inclusion in our study questionnaire. By completing the sentence, the participant expresses a positive or negative evaluation of the belief statement [52]. The bipolar endpoints were expressed as a 7-point Likert scale namely, 1 = strongly disagree, 2 = disagree, 3 = disagree somewhat, 4 = neither agree nor disagree, 5 = agree somewhat, 6 = agree, 7 = strongly agree. Using the Likert scale allows the evaluation of the strength of the belief within the target population by calculating the mode of each belief.

### 2.4. Data Analyses

STATISTICA version 13.3 [54] and STATA 15 [55] were used to clean and analyse the data. The data were tested for normality using Shapiro Wilks tests (*p* > 0.05 = normal). Data with a normal distribution were described using means and standard deviations (SD). Medians and inter-quartile ranges (IQR) were used for data with a non-normal distribution. For ease of comparison to other studies [21,22,23,24,25,26,27,28,29], both median(IQR) and mean(SD) were recorded for dietary intake of energy, macronutrients and micronutrients. Categorical data were described using frequencies and percentages. Belief statements were expressed using mode values. Spearmen correlation co-efficient and their *p*-values were computed to test associations between beliefs and the food group intakes. Univariate logistic regression analyses were done to explore the associations between sociodemographic factors and health related perceptions with selected dietary variables (macronutrients as a percentage of TE as well as sugar, SSBs and F&V intake). To create dichotomous dietary variables to divide the group in those who met and did not met a dietary recommendation, the following cut-points were used: sugar 0 g, SSBs 0 mL and total F&V 400 g per day. Practical cut-points for macronutrient intake as a % of TE were used namely: 45% for carbohydrates, 15% for protein and 35% for fats. Variables with *p*-values < 0.1 at univariate analysis, were included in a forward stepwise multivariate logistic regression. If the food group had no variables that were significant at *p* < 0.1, no multivariate regression was carried out. Variables were manually added to the logistic regression, with the binary outcome of reaching recommended intake (yes/no) for each of the major food groups. Variables tested for association were socio-demographic variables, GA, perception of food choices and physical activity, self-reported reproductive health and the hospital they were treated in. A *p*-value of <0.05 was considered significant and 95% confidence intervals (CIs) were reported for all odds ratios (ORs).

## 3. Results

### 3.1. Socio-Demographic History and Pregnancy History

The mean (SD) age of the women was 32.2 (5.3) years and the mean gestational age was 33.0 (3.4) weeks. Table 2 shows that just more than one-third of the women had an advanced maternal age of ≥35 years. The majority, (73.6%), of the participants was recruited from GSH. Half of the sample (58.9%) was mixed-race ancestry, 34.7% were Black and the remaining 6.5% were either White or Indian. The majority of the sample had an LSM score between 6 and 9 and 64.4% reported that their food choices are ‘mostly healthy.’ Most participants (97.5%) were willing to participate in a wellness program should one be available and the preferred means of communication were evenly distributed between one-on-one, group sessions and social media (Table 2).

### 3.2. Dietary Intake

The mean daily energy intake was 7268 KJ (Table 3). As for fibre intake, 80.9% consumed below the recommended 28 g/day. For the micronutrients, a high percentage of the sample had inadequate intakes of vitamin D (87.4%), folate (96.5%) and iron (91.3%), which are particularly important micronutrients in pregnancy. 

Table 4 shows the macronutrients as a percentage of total energy and sugar, SSBs and F&V intake. The median (IQR) amount of added sugar and SSBs was 4.0 g (0.00–12.5) and 17.9 mL (0.00–132.8) per day, respectively. A quarter (25.1%) consumed more than one small glass of SSBs per day, one third (33.5%) had more than two teaspoons of sugar per day and only 31.4% of the sample consumed the recommended 400 g or more of F&V daily.

### 3.3. Beliefs Relating to the Intake of Sugary Foods and Drinks and Fruits and Vegetables

For all the behavioural, control and normative belief statements relating to F&V intake the mode was 6 indicating that the participants agree with the statements (Table 5). Agreement with behavioural beliefs indicated that participants were aware of the health benefits of eating more F&V such as feeling better physically and assisting weight loss. Agreement with the normative beliefs shows that participants believed they had support from family or peers to consume more F&V. Agreement with the control beliefs indicated the participants’ perceived ease/difficulty in consuming more F&V.

Beliefs relating to sugar intake had a mode of 6, except that participants disagree (mode = 2) with the statement that “low sugar/sugar-free foods taste good or are tasty,” indicating a perceived difficulty to consuming low sugar/sugar-free foods.

There were significant positive correlations, albeit weak, between dietary intake of F&V and the beliefs that F&V make you feel better physically (*r* = 0.16, *p* = 0.0176) as well as F&V are easy to find in shops nearby (*r* = 0.15, *p* = 0.0221), (Table 5). Significant negative correlations were found between dietary intake of sugar or SSBs and the beliefs that it was easy to exclude sugary foods/snack/drinks in ‘their’ daily diet (sugar: *r* = −0.259, *p* < 0.001; SSBs: *r* = −0.246, *p* < 0.001), knowing how to control cravings would make it easier to eat less sugary foods/snacks/drinks (sugar *r* = −0.153 *p* = 0.0205; SSBs *r* = −0.152 *p* = 0.0209) and low sugar/sugar-free foods/snacks/drinks taste good (sugar *r* = −0.271 *p* < 0.001; SSBs *r* = −0.129 *p* = 0.0498). Significant negative correlations were also evident between dietary intake of sugar but not SSBs and the beliefs that eating less sugary foods/snacks/drinks will help reduce the risk of disease such as diabetes (*r* = −0.184, *p* = 0.0049) and eating/drinking less sugary foods/snacks/drinks is up to themselves (*r* = −0.149 *p* = 0.02).

### 3.4. Univariate Association Analyses

Table 6 shows the results of univariate logistic regression analysis for associations between dietary intake variables (sugar, SSB and protein as a % of TE) and sociodemographic or health related variables. Each yearly increase in age of participants significantly increased the likelihood of them meeting the recommendation for SSBs (0 mL) by 6% (OR, 1.06, 95% CI, 1.01–1.11, *p* = 0.029) and meeting a lower intake of protein (≤15% of TE) by 9% (OR, 1.09, 95% CI, 1.03–1.14, *p* = 0.002). Participants from MMH were 77% (OR, 0.33, 95% CI 0.17–0.64, *p* = 0.001), 91% (OR, 0.09, 95% CI, 0.03–0.25, *p* < 0.001) and 61% (OR, 0.39, 95% CI, 0.20–0.73, *p* = 0.003) less likely to meet the recommended amount of SSBs (0 mL), sugar (0 g) and lower intake of protein (≤15% of TE) respectively compared to those from GSH. Participants who self-rated their food choices as ‘mostly unhealthy’ were 86% (*p* = 0.024), 84% (*p* = 0.027) and 93% (*p* = 0.015) less likely to meet the recommended amount of SSBs (0 mL) and sugar (0 g) and lower intake of protein (≤15% of TE) respectively compared to those who self-reported their food choices as ‘mostly very healthy’ (Table 6). Each additional child that a participant had increased their likelihood of meeting the recommendation for SSBs (0 mL) by 29% (OR 1.29, 95% CI, 1.04–1.59, *p* = 0.020).

Race, gestational age, LSM score and parity were not associated with meeting recommended intake of table sugar, SSBs and 15% protein of TE. There were no significant associations between other dietary intake variables (F&V, 35% fat and 50% carbohydrates of TE) and the sociodemographic or health related variables (results not reported in Table 6).

### 3.5. Multivariate Association Analyses

The univariate associations between SSBs and age or number of children were not significant in the multivariate logistic regression (Table 7), while being treated at GSH versus MMH (OR 0.41, 95% CI 0.19–0.86, *p* = 0.013) and having the perception of consuming a “mostly very healthy diet” compared to those indicating “mostly unhealthy” diet (OR 0.16, 95% CI 0.03–0.98, *p* = 0.048) remained significant predictors of meeting the recommended 0 mL SSBs per day. In the multivariate analyses for meeting the cut-point of protein intake as a % of TE, the treatment hospital was the only variable that remained significant (Table 7), while no significant associations in the multivariate analyses were found for meeting the recommended sugar intake (0 g) (results not included in the table).

## 4. Discussion

The dietary intake of the pregnant women with GDM in this study was not optimal and fell short of several nutritional guidelines. The total energy intake (7268 kJ) was lower than the 11,844 kJ (2830 kcal) recommended by the Institute of Medicine (IOM) of the United States (US) [34] for a normal weight female in third trimester of pregnancy. However, energy requirements during pregnancy remain controversial and are influenced by several factors, including pre-gestational weight status, gestational weight gain and fat deposition [58]. The IOM recommendation is based on healthy active Americans and Canadians at the reference height and weight and does not necessarily reflect the energy requirements of the woman in our sample. The most recent guidelines of the American Diabetes Association [15] indicate that the optimal energy requirements of women with GDM are unknown and no research could be traced that investigated whether their energy requirements are different to a pregnancy without GDM. Javonovic [59] reported that the energy requirements for obese women with GDM may be much lower, as preliminary studies indicated that diets between 6300 kJ (1500 kcal) and 7560 kJ (1800 kcal) resulted in improved fasting and mean daily glucose levels, without the development of ketonemia. As national surveys indicated that 64% [60] and 62.2% [61] of South African women aged 15 and older are overweight or obese, it is possible that such lower energy requirements reflect the needs of the majority of women in our sample. With a dearth of comparable studies on GDM women, our energy intake was slightly lower than the energy intakes of 7677.2 kJ (1834 kcal) [24] 8425.7 kJ (2013.6 kcal) [26] and 9123 kJ (2180 kcal) [29] previously reported for non-diabetic pregnant South African women. Women with hyperglycaemia in pregnancy at MMH and GSH are referred to the hospital dietitian for a one-on-one counselling session and/or they receive dietary information from the diabetes educator or nurse. At the time of administering the questionnaire, most participants had previous dietary counselling. It is possible that they might have changed their diet after counselling, or they might have been inclined to report more favourably on their intake. As energy requirement are not calculated during consultations, portion control of energy-dense foods would remain an essential strategy during dietary counselling sessions.

The macronutrient distribution as a % of TE was not optimal if evaluated against the 2017 SEMDSA guidelines [1] that recommend carbohydrates to be up to 40% of TE, 40% of TE from fats and 20% TE from protein as well as the other international associations recommending carbohydrates < 50% of TE for GDM women (Table 1). In a country where food insecurity is high and carbohydrate rich foods such as maize meal, white rice and bread are the staple diet [62], the macronutrient distribution of the women in this study (55% for carbohydrates and 14.5% for protein) could be expected. Research in the form of RCTs is needed to establish whether this type of macronutrient distribution is problematic for optimal glycaemic control in women with GDM in South Africa. There was however no significant association found between carbohydrate or protein intake and LSM.

When comparing the actual grams of carbohydrate, protein and fat to other studies that were conducted on non-GDM pregnant women in South Africa, it is noted that the mean carbohydrate intake of our participants (220 g ± 104.5 g) was similar to the 230 g (SD ± 57.5) reported by Cormick et al. [24] but slightly less than the 292.45 ± 72.2 g by Kesa and Oldewage-Theron [26] and 334.7 g reported for pregnant adolescents by Tshitaudzi [29]. The protein intake (60.3 ± 27.5 g) was in line with the other South African studies namely, 54.7 ± 7.8 g by Cormick et al. [24], 60.9 g by Tshitaudzi [29] and 73.2 ± 23 g by Kesa and Oldewage-Theron [26]. However, total fat intake of 67.7 g (±44.2 g) was slightly higher than previous studies namely 59.1 ± 6.4 g [24], 62.3 ± 23.7 g [26] and 48.4 g in Tshitaudzi [29]. For optimal blood glucose control and general health purposes during pregnancy, it is important to consider the type of carbohydrates consumed. It is recommended that carbohydrate intake should consist mostly of vegetables, legumes, fruits, dairy and whole grains [15] and consuming adequate amounts of these foods will result in sufficient intake of dietary fibre. In our sample 80% of participants were below the recommended 28 g of fibre per day, similar to the findings of Tshitaudzi [29] and Mostert et al. [28] in pregnant South African women. Fibre is an important dietary component during pregnancy as it is beneficial to prevent constipation and haemorrhoids and assists in stabilizing blood glucose levels. Louie [19] reported that a high fibre diet in women with GDM was significantly associated with a lower prevalence of LGA, macrosomia and emergency caesarean section compared to a low glycaemic index diet.

F&V are an essential source of dietary fibre but only 31.4% of participants met the WHO/FAO [21] recommendation of 400 g or more per day. The low F&V intake could possibly be due to the lack of affordable options in close vicinity of the households [63]. In line with this, our participants who had higher F&V intakes strongly held the belief that these items are easy to find in shops close to them and that they make you feel better physically. However, none of the sociodemographic factors tested, including LSM, were associated with the consumption of F&V. It is possible that the LSM were not sensitive enough to discriminate between SES of participants or that participants might not be aware of the WHO/FAO recommendation of 400 g of F&V and how to incorporate it in their daily diet. This may be an area to address in dietary intervention in this population. Participants agreed that F&V are affordable and do not take a long time to prepare, therefore it is likely that unavailability is hindering intake. Interventions aiming at increasing F&V consumption in this population could encourage consuming seasonal F&V and planting their own vegetables at home or developing community gardens.

SSBs are a source of high glycaemic carbohydrates. The intake of refined carbohydrates in the form of SSBs is strongly discouraged for optimal blood glucose control [15]. In our sample of pregnant women, 63% reported drinking up to half a small glass (125 mL) of SSBs daily. A higher intake of SSBs was found in younger women and those who self-reported that their diet consists mostly of ‘unhealthy food choices.’ Those who consumed more SSBs believed that it was not easy to exclude sugary foods/drinks from their diet and that low sugar/sugar free food/drinks do not taste good. Therefore, focus is required on addressing healthy alternatives to SSBs which taste good and satisfy cravings. Practical ideas may include fruit infused water or recipes for homemade ice tea and awareness of the equivalent number of spoons of table sugar present in SSBs.

Another source of glycaemic carbohydrates is table sugar. Only 34.7% of our participants reported not adding sugar to hot beverages or porridge. A higher consumption of table sugar was significantly associated with women attending MMH and those who self-reported that their diet consists of ‘mostly unhealthy’ food choices. As women with high risk pregnancies and poorly controlled blood glucose levels are treated at GSH, it is possible that more emphasis has been placed on the role of diet on blood glucose levels in women receiving care there than with women attending the secondary community based hospital (MMH). This could be due to receiving a higher number of inputs on changes required from various health professionals when treated at a tertiary hospital. Mothers having had GDM in a previous pregnancy may have been more cautious and have applied their knowledge from previous experience. There were more significant associations between beliefs and table sugar intake, than with the intake of SSBs. For instance, a lower sugar intake was significantly associated with agreement with five beliefs including eating less sugar will reduce their risk of disease such as diabetes, eating less sugary foods/snacks/drink is easy, low sugar foods/snacks/drinks are tasty and that eating less sugar was up to them. This may indicate that there is a stronger awareness of or emphasis on the impact of added table sugar in glycaemic control than with SSBs. The participants also believed that knowing to control ‘their’ cravings for sugary foods/snacks/drinks during pregnancy will make it easier for them to eat less of these foods. Sweet cravings have been reported to appear in late GDM pregnancy [64]. Different theories exist to explain food cravings in pregnancy, one of which is ‘nutrient deficits’ [65].

Many participants’ micronutrient intake fell short of the recommendations in pregnancy, in keeping with findings of poor micronutrient intakes in pregnant women in South Africa [22,24,28]. The majority of the GDM participants fell below the recommendations for iron, calcium and folic acid which are essential micronutrients in pregnancy. However, if participants were underreporting, as found in South African population [66] it is likely that the reported micronutrient levels are not a true reflection of the participants’ actual micronutrient intake. On the other hand, with the intake of energy-dense, nutrient poor foods such as SSBs and added table sugar and a low intake of F&V, the finding that the micronutrient intake was inadequate may be plausible. Several micronutrients are especially important for GDM women. For example: iron plays an important role in pregnancy and blood glucose metabolism [67] and calcium plays a critical role in muscle contraction and in glucose uptake by cells [68]. Vitamin D deficiency is common during pregnancy even in countries with sunny climates, as South Africa and is associated with an increased risk of developing pre-eclampsia [69] and GDM [70]. Dairy products and dark green leafy vegetables can be encouraged as a source of calcium and vitamin D. The mean dietary folate intake of the participants (excluding supplements) was 244.8 micrograms, which fall well below the recommended 600 micrograms. However, all pregnant women in the public-sector health service are given a folic acid supplement of 5 mg a day, which far exceeds the increased requirements of 600 micrograms during pregnancy. While supplementation of folate in pregnancy is given to reduce the chances of neural tube defects, a recent study by Zhu [71] illustrated that this daily supplementation may increase the risk of. This is therefore a possible cause for concern and the policy in South Africa and elsewhere may need reviewing. However, further evidence of the potential adverse effects of excess folate in pregnancy is still needed and for now the benefits of folate supplementation during pregnancy outweigh the potential risks of excess folate during pregnancy.

This study may serve as evidence to plan effective nutrition education programmes that promote lifestyle changes in pregnant women with hyperglycaemia first detected in pregnancy. With 97.5% of participants responding positively to participating in a wellness programme, it indicates that this population has a desire to receive information and support to change their lifestyle habits. These women would be good candidates for a lifestyle intervention that could be delivered using a combination of methods such as one-on-one counselling, groups session, printed materials and social media platforms.

This study is limited by the fact that all variables were self-reported, which may not necessarily reflect the true situation. We aimed to improve the validity of these outcome measures by going through rigorous processes to ensure a well-developed questionnaire. We did not use an objective method to verify energy and nutrient intake nor another dietary intake method such as a 24-h recall to validate the FFQ results. It may be likely that participants under-reported their dietary intake to impress the fieldworker by not admitting ‘unhealthy foods’ or due to memory gaps common when recalling items over the last 2 weeks. Underreporting is common in obese populations [72,73], however this has not been investigated in GDM. Current body weight was not measured; however, pre-pregnancy weight and BMI classification would be the ideal measures for the estimation of caloric requirements. The picture-sort method used with the FFQ, improves ease and accuracy of response in diet recall [74]. As the number of participants that were classified in some categories of sociodemographic variables were small, the sample size restricted statistical power of univariate and multivariate analyses.

## 5. Conclusions

This study found that the macronutrient distribution in pregnant women with GDM in Cape Town did not meet the dietary guidelines of local and international associations as carbohydrate intake are high and protein and fat intakes are low. Inadequate amounts of dietary fibre, F&V and key micronutrients important for pregnancy were consumed and the intake of added table sugar and SSBs were too high in our population. We recommend that interventions be developed and tested for women with GDM in Cape Town to: (1) establish the macronutrient distribution required for optimal glucose control, (2) meet the recommendations of 400 g/d for F&V and 28 g/d for fibre, (3) limit intake of added table sugar and SSBs and (4) improve the nutrient density of the diet so that adequate intakes of micronutrients are ensured. This study contributes to new knowledge as it investigated the diet of GDM women in Cape Town, which had not previously been done. The study reveals beliefs underlying the dietary intake of F&V, table sugar and SSB. The strongly held beliefs regarding sugary foods/drinks may contribute to poor adherence to nutritional guidelines.

## Figures and Tables

**Table 1:** Dietary recommendation in Gestational Diabetes from different associations.

Macronutrients	SEMDSA (2017) [1]	ADA (2007) [31]	Fourth International Workshop-Conference on Gestational Diabetes Mellitus, 1998 [32]	CDA (2006) [33]	FAO *(2002)/IOM * [34]
Energy		1500–2800/day +340 kcal 2nd trimester +452 kcal 3rd trimester	25 kcal/kg body weight		+85 kcal 1st trimester +285 kcal 2nd trimester +475 kcal 3rd trimester
Carbohydrates	40% carbohydrate (complex, low-glycaemic index, high fibre)		35–45% of total calories	45–50% TE	45–65% TE At least 175 g/day
Added sugars	<5% total energy			<10%	<25% TE
Protein	20% protein		protein 20–25%		10–25% At least 71 g/day
Total Fats	40% fat (at least 50% unsaturated)		fat 35–40%	Up to 40% TE	20–35% TE

SEMDSA, Society for Endocrinology, Metabolism and Diabetes of South Africa; ADA, American Diabetes Association; CDA, Canadian Diabetes Association; FAO, Food and Agricultural Association; IOM, Institute of Medicine. * Recommendations for normal pregnancy.

**Table 2:** Sociodemographic profile and pregnancy history of sample (*n* = 239).

Variable	Categories	*n*	Percentage of Total Sample *n* = 239 (%)
**Recruitment hospital**	GSH	176	73.6
	-MMH	63	26.4
**Age**	<35 years	154	64.4
	≥35 years	85	35.5
**Gestational Age**	<33 weeks	108	45.2
	≥33 weeks	131	54.8
**Race**	Black	83	34.7
	White	3	1.3
	Indian	6	2.5
	Mixed-race ancestry *	141	58.9
	Other	6	2.5
**Living Standard Measurement**	LSM ≤ 4	6	2.5
	LSM 5–7	95	39.8
	LSM 8–10	138	57.8
**Number of children**	0	55	23.0
	1	67	28.0
	2	68	28.4
	3–6	49	20.5
**Parity**	1st	39	16.3
	2nd	62	25.9
	3rd	78	32.6
	4th	35	14.6
	5th to 10th	25	10.6
**GDM in previous pregnancy**	Yes	50	20.9
	No	150	62.7
	N/a if 1st pregnancy	39	16.3
**What do you think of the food choices you make most of the time? (4 or more times per week)**	Most very healthy	9	3.7
	Mostly healthy	154	64.4
	Mostly unhealthy	66	27.6
	Mostly very unhealthy	10	4.1
**If a wellness program was available for pregnant women, would you enrol?**	Yes	233	97.5
	No	6	2.5
**What is the preferred way in which you like to receive information on health and nutrition?**	One-on-one	64	26.7
	Group session	71	29.7
	Print material	38	15.9
	Social media	60	25.1

GSH: Groote Schuur Hospital, MMH: Mowbray Maternity Hospital, LSM: living standards measure, GDM: gestational diabetes mellitus. * Mixed-race ancestry: this population group in South Africa are also referred to as the Cape Coloureds in Cape Town and has a mixed ancestry with genetic material mainly from Khoisan, Bantu African, Northern European, South Asia and South-East Asia origins.

**Table 3:** Mean and adequacy of macronutrients, vitamins and mineral intake per day by the sample (*n* = 230).

Energy and Nutrients	Mean (SD)	Median (IQR)	Guideline (Cut Point)	Percentage of Sample That Fell below Cut Point (%)
**Total energy (KJ)**	7268.0 (3527.5)	6437.9 (4863.3–8687.7)		
**Protein (g)** **(%TE)**	60.3 (27.5) 14.7 (3.4)	55.0 (41.4–70.8) 14.6 (12.5–16.9)	71 g/day ^a^* 20% TE ^b^	77.4 93.5
**Total fat (g)** **(%TE)**	67.7 (44.2) 33.1 (7.9)	58.2(38.8–82.1) 31.8 (28.0-37.9)	40% TE ^b^	80.4
**MUFA (g)** **(%TE)**	23.9 (16.6) 11.0 (3.4)	19.9 (13.2–27.5) 10.6 (8.5–13.1)	≤20% TE ^c^	97.8
**PUFA (g)** **(%TE)**	17.6 (14.6) 8.0 (3.8)	13.4 (8.5–21.4) 7.1 (5.7–9.7)	≤10% TE ^c^	78.2
**Saturated fat (g)** **(%TE)**	20.1 (14.1) 9.8 (3.0)	17.2 (11.1–24.0) 9.6 (7.9–11.5)	<7% TE ^c^	14.7
**Cholesterol (g)**	265.6 (243.2)	194.9 (121.3–310.1)	<200 mg ^c^	52.1
**Carbohydrates (g)** **(% TE)**	220.0 (104.5) 53.0 (8.6)	197.4 (142.9–270.4) 53.6 (47.4–58.7)	135 g/day ^a^ 40% TE ^b^	21.7 7.8
**Fibre (g)**	21.7 (11.3)	20.0 (14.8–26.4)	28 g ^a^	80.9
**Alcohol (g)**	0.019 (0.2)	0.00 (0.00–0.00)		
*FAT SOLUBLE VITAMINS*				
**Vitamin A (mcg)**	1058.2 (645.4)	877.3 (598.7–1396.5)	550 mcg/day ^a^	20.4
**Vitamin D (ug)**	5.5 (5.1)	4.0 (2.4–6.6)	10 ug/day ^a^	87.4
**Vitamin E (mg)**	13.4 (10.0)	10.9 (7.0–16.1)	12 mg/day ^a^	60.0
*WATER SOLUBLE VITAMINS*				
**Thiamin (mg)**	1.3 (0.7)	1.2 (0.9–1.6)	1.2 mg/day ^a^	30.9
**Riboflavin (mg)**	2.0 (1.5)	1.5 (1.0–2.4)	1.2 mg/day ^a^	25.2
**Niacin (mg)**	21.7 (11.4)	19.2 (14.9–26.9)	14 mg/day ^a^	30.4
**Vitamin B6 (mg)**	2.9 (1.6)	2.7 (1.8–3.7)	1.6 mg/day ^a^	6.5
**Vitamin B12 (mcg)**	4.5 (4.2)	3.1 (2.1–5.2)	2.2 mcg/day ^a^	21.7
**Pantothenate (mg)**	4.5 (2.3)	4.0 (2.9–5.7)	6 mg/day ^a^	80.0
**Biotin (mcg)**	34.7 (19.4)	30.9 (22.9–42.1)	30 mcg/day ^a^	47.8
**Folate (ug)**	244.8 (149.8)	218.2 (154.2–291.5)	520 ug/day ^a^	96.5
**Vitamin C (mg)**	97.4 (124.7)	61.5 (36.2–124.7)	70 mg/day ^a^	56.5
*MINERALS*				
**Calcium (mg)**	651.9 (402.7)	561.1 (379.2–789.7)	800 mg/day ^a^	75.6
**Iron (mg)**	13.4 (8.0)	11.6 (9.0–15.7)	22 mg/day ^a^	91.3
**Magnesium (mg)**	251.5 (128.3)	231.2 (177.8–296.2)	290 (19–30 y) 300 (31–50 y) mg/day ^a^	74.3
**Phosphorus (mg)**	1005.8 (491.7)	902.7 (672.6–1198.1)	580 mg/day ^a^	16.1
**Potassium (g)**	2038.2 (945.1)	1881.1 (1400.4–2376.7)	4.7 g/day ^a^	98.3
**Sodium (mg)**	1741.5 (944.0)	1531.8 (1138.8–2079.2)	1500 mg/day ^a^	48.3
**Zinc (mg)**	10.3 (4.5)	9.7 (7.3–12.2)	9.5 mg/day ^a^	42.6
**Copper (ug)**	1.1 (0.6)	1.0 (0.7–1.3)	800 ug/day ^a^	32.6
**Manganese (mg)**	2.2 (1.3)	1.9 (1.3–2.9)	2.0 mg/day ^a^	53.9

* excludes dietary data< 2092 KJ and >20,920 KJ. Data was non-normally distributed, thus Median (IQR) applies. Mean (SD) was included to compared with results from previous studies. ^a^ EAR or AI (when EAR is not available), ^a^* RDA (of the DRI) [56], ^b^ SEMDSA 2017 [1]. ^c^ TLC guidelines by National Heart, Lung and Blood Institute [57]. TE: total energy, MUFA: mono-unsaturated fatty acids, PUFA: poly-unsaturated fatty acids, EAR: estimated average requirements, AI: adequate intakes, DRIs: dietary reference intakes, TLC: Therapeutic Lifestyle Changes.

**Table 4:** Breakdown of the macronutrient distribution of the sample and the percentage of participants’ macronutrients intake as percent of total energy and intake of table sugar, sugar sweetened beverages (SSBs) and fruits and vegetables (F&V).

Macronutrients as a % of TE and Food Categories	Percentage of Total Group (*n* = 230) (%)
**Carbohydrates (% Total energy)**	
<40	7.8
40–44.9	12.1
45–50	13.9
>50	66.0
**Protein (% Total energy)**	
<10	7.4
10–15	47.8
15.1–20	38.3
>20	6.5
**Fat (% Total energy)**	
<30	35.2
30–34.9	25.2
35–40	20.0
>40	19.5
Teaspoons sugar *	
0 tsp	34.7
less than or equal 2 tsp	31.8
more than 2 tsp	33.5
SSBs (small glasses) **	
Up to ½ small glass	63.6
½ to 1 small glass	11.3
More than 1 small glass	25.1
**Fruits and Vegetables**	
Less than 200 g	28.9
Between 200 g and 400 g	39.3
400 g and more	31.4

* level teaspoons sugar (5 g) added to tea/coffee, breakfast cereals/porridge ** 1 small glass = 125 mL.

**Table 5:** Beliefs associated with the intake of Fruits and Vegetables and Sugar.

Beliefs Related to Fruit and Vegetable	Belief Type	Mode	Frequency of Mode (%)	Correlation of Belief with F&V Intake * (rho, *p*-Value)	
Eating fruits and vegetables every day will make me feel better physically.	Behavioural	6.0	49.8	0.159 (0.017)	
Eating fruits and vegetables every day will help control my weight.	Behavioural	6.0	53.9	0.081 (0.2212)	
Eating less fruit will help control my blood sugar levels (i.e., to reduce the risk of diabetes).	Behavioural	6.0	37.6	−0.036 (0.582)	
Vegetables do not take a long time to prepare.	Control	6.0	34.3	0.019 (0.765)	
Fruits and vegetables are affordable.	Control	6.0	45.6	0.029 (0.661)	
Fruits and vegetables are easy to find in the stores/shops nearby.	Control	6.0	54.8	0.151 (0.022)	
I am confident that I can eat the recommended amount of fruits and vegetables every day.	Control	6.0	44.7	0.101 (0.124)	
Most people who are important to me eat fruits and vegetables every day.	Normative	6.0	38.9	0.052 (0.431)	
**Beliefs related to sugar**	**Belief type**	**Mode**	**Frequency of mode (%)**	Correlation of belief with sugar intake * (rho, *p*-value)	Correlation of belief with SSB intake * (rho, *p*-value)
Eating less sugary foods/snacks/drinks will help reduce the risk of diseases e.g., diabetes.	Behavioural	6.0	51.5	−0.184 (0.005)	−0.109 (0.098)
It is also important to limit my intake of sugary foods/snacks/drinks after the pregnancy.	Behavioural	6.0	60.7	−0.175 (0.007)	0.028 (0.672)
Decreasing the amount of sugary foods/snacks/drinks I eat will help control my weight.	Behavioural	6.0	54.0	−0.004 (0.945)	−0.064 (0.337)
Increasing the amount sugary foods/snacks/drinks I eat and drink make me feel unwell (tired, headache, dizzy, signs of hyper glycaemia, etc.).	Behavioural	6.0	44.4	−0.021 (0.743)	0.063 (0.335)
I want to reduce the amount of sugary foods/snacks/drinks I eat and drink to prevent pregnancy/birth complications.	Behavioural/Control	6.0	53.6	−0.055 (0.406)	−0.016 (0.805)
It is easy to exclude sugary foods/snacks/drinks from my daily diet.	Control	6.0	33.2	−0.259 (<0.001)	−0.246 (<0.001)
Foods/snacks/drinks that are low sugar/sugar free are easy to find in my surroundings.	Control	6.0	39.9	−0.022 (0.736)	−0.069 (0.292)
Eating/drinking less sugary foods/snacks/drinks is up to me.	Control	6.0	56.1	−0.149 (0.023)	−0.128 (0.052)
Knowing how to control my cravings for sugary foods/snacks/drinks during pregnancy will make it easier for me to eat less of these foods.	Control	6.0	54.6	−0.153 (0.021)	−0.152 (0.021)
Low sugar/sugar-free foods/snacks/drinks are expensive.	Control	6.0	42.3	0.011 (0.859)	−0.002 (0.975)
Low sugar/sugar-free foods taste good/are tasty.	Control	2.0	27.3	−0.271 (<0.001)	−0.129 (0.049)
People around me eat/serve sugary foods/snacks/drinks at most events/functions (social, religious, or work events)	Normative	6.0	46.9	−0.031 (0.641)	0.061 (0.356)

* Spearman Rank Order Correlation; Behavioural beliefs: are the perceived consequences (positive or negative) of the behaviour; Control beliefs: are factors that facilitate or hinder the behaviour; Normative beliefs: extent to which other people are important to them think they should or should not perform a certain behaviour [50].

**Table 6:** The Univariate association between sociodemographic factors and the proportion of participants consuming the recommended intakes of SSBs, added sugar and Protein.

Variables	SSBs			Added Sugar			% Protein		
Recommendation	0 mL			0 g			15% of Total Energy		
	OR	95% CI	*p*-value *	OR	95% CI	*p*-value *	OR	95% CI	*p*-value *
**Age**	1.06	1.01–1.11	0.029	1.02	0.97–1.08	0.353	1.09	1.03–1.14	0.002
**Gestational age**	0.88	0.52–1.49	0.598	0.98	0.90–1.06	0.553	0.95	0.87-1.02	0.164
**Hospital GSH versus MMH**	0.33	0.17–0.64	0.001	0.09	0.03–0.25	0.000	0.39	0.20–0.73	0.003
**GDM in previous pregnancy**	0.68	0.43–1.06	0.085	0.51	0.27–1.0	0.049	0.61	0.32-1.17	0.138
**Race**									
**Black versus**									
**White**	3.4	0.29–39.1	0.326	0.73	0.06–8.35	0.798	0.46	0.04–5.32	0.528
**Indian**	0.85	0.15–4.92	0.856	0.29	0.03–2.61	0.270	0.46	0.08–2.68	0.391
Coloured **	1.46	0.83–2.57	0.186	0.69	0.39–1.22	0.199	0.67	0.38–1.16	0.152
**LSM**	0.99	0.90–1.09	0.921	0.95	0.56–1.05	0.310	1.03	0.94–1.14	0.531
**Self-reported food choice**									
**“Mostly very healthy** **” versus**									
**Mostly healthy**	0.36	0.07–1.94	0.236	0.51	0.11–2.35	0.387	0.16	0.02–1.36	0.094
**Mostly unhealthy**	0.14	0.02–0.77	0.024	0.16	0.03–0.81	0.027	0.07	0.01–0.59	0.015
**Mostly very unhealthy**	0.2	0.23–1.71	0.142	0.6	0.08–4.40	0.615	0.21	0.02–2.52	0.217
**Self-reported physical activity level**									
**“very inactive” versus**									
**Inactive**	1.07	0.38–3.01	0.896	0.54	0.18–1.57	0.263	1.15	0.40–3.12	0.790
**Active**	1.04	0.39–2.72	0.936	0.88	0.33–2.31	0.797	1.61	0.60–4.30	0.341
**Very active**	1.25	0.36–4.26	0.721	0.85	0.24–2.98	0.809	2.68	0.76–9.37	0.122
**No of children**	1.29	1.04–1.59	0.020	1.14	0.92–1.41	0.224	1.14	0.92–1.40	0.225
**Wellness program—Yes versus**									
**no**	2.87	0.51–15.9	0.229	3.89	0.69–21.7	0.121	1		

* Manual logistic regression, with the binary outcome of reaching recommended intake (yes/no) for each of the major food groups. Variables with *p*-values less than 0.1 at univariate analysis, were included in the multivariate regression (forward stepwise). If the food group had no variables that were significant at *p* < 0.1, no multivariate regression was carried out. ** mixed-race ancestry: this population group in South Africa are also referred to as the Cape Coloureds in Cape Town and has a mixed ancestry with genetic material mainly from Khoisan, Bantu African, Northern European, South Asia and South-East Asia origins. CI: Confidence interval; OR: Odds Ratio.

**Table 7:** Multivariate logistic regression analyses between sociodemographic factors and the proportion of participants consuming the recommended intake of SSBs (0 mL) and a Protein intake ≤ 15% of TE.

Variables	SSBs			% Protein		
	OR	95% CI	*p*-Value *	OR	95% CI	*p*-Value *
**Age**	1.03	0.97–1.10	0.312	1.05	0.99–1.11	0.068
**Hospital GSH versus**						
**MMH**	0.41	0.19–0.86	0.019	0.36	0.18–0.74	0.005
**GDM** **in previous pregnancy**	0.67	0.32–1.39	0.279	-	-	-
**Self-reported food choice**						
**“Mostly very healthy” versus**						
**Mostly healthy**	0.34	0.06–1.97	0.230	0.37	0.06–2.17	0.277
**Mostly unhealthy**	0.16	0.03–0.98	0.048	0.17	0.03–1.06	0.058
**Mostly very unhealthy**	0.19	0.02–1.80	0.149	0.20	0.02–1.89	0.162
**No of children**	1.22	0.92–1.62	0.161	-	-	-

* Forward stepwise multivariate logistic regression analyses with the binary outcome of reaching recommended intake (yes/no) for SSBs of 0 mL and the cut-point for protein of 15% of TE.

## Supplementary Materials

The following are available online at http://www.mdpi.com/2072-6643/10/9/1183/s1.
