# Supplementary material for: Dietary Intake and Beliefs of Pregnant Women with Gestational Diabetes in Cape Town, South Africa
Source: Nutrients. 2018 Aug 28;10(9):1183. doi: 10.3390/nu10091183 (PMC6164942; doi:10.3390/nu10091183)
Supplement: Supplementary file 1 [file nutrients-10-01183-s001.pdf]

Inclusion criteria:
18 years or older
≥28 week gestation
GDM or IGT
[illegible]

GDM Study

Phase 2: Questionnaire (Cross-sectional Survey)

Patient sticker

Contact details:

Participant code: …………………

Age (years): ________

Weeks' gestation: ________

Expected due date: ________________

Race:

Black	White	Indian	Coloured	

Pregnancy:

1st	2nd	3rd	4th	5th

No of children:

0	1	2	3	4	5

Section A		
**Please tick next to the correct answer**		
**1.** Did you have diabetes with your previous pregnancy?	Yes	
	No	
	N/A	
**2.** How would you rate your health so far during this pregnancy?	Excellent	
	Good	
	Fair	
	Poor	
**3.** How happy were you with your weight before pregnancy? Tick	Happy	
	Somewhat happy	
	Unhappy	
**4.** Do you think your weight gain during this pregnancy is:	Too little	
	Just right	
	Too much	
**5.** How would you rate your current level of physical activity:	Very inactive	
	Inactive	
	Active	
	Very active	
**6.** What do you think of the food choices you make most of the time (on 4 or more times per week):	Mostly very healthy	
	Mostly healthy	
	Mostly unhealthy	
	Mostly very unhealthy	
**7.** Do you suffer from high blood pressure?	Yes	
	No	

**8.** How many fruit do you eat (1 fruit = 1 med or 2 small)	Per day OR	
	Per week	
**9.** How many vegetables do you eat (1 veg = ½ cup)	Per day OR	
	Per week	
**10.** If a wellness programme was available for pregnant women, would you enrol?	Yes	
	No	
**11.** What is the preferred way you would like to receive information on health/ nutrition?	One-on-one session/ individual	
	Group session	
	Print material (poster, pamphlet, booklets etc.)	
	Social media (internet, sms etc.)	

Section B: LSM calculation

NB! Please tick the items in the table below that you have in your household

ITEM	tick	ITEM	tick
House/ property worth (250 000) or more		Dishwashing machine	
DVD Player/ Blu Ray player		Tumble dryer	
Living in a non-urban area		Home security service	
Refrigerator or combined fridge/ freezer		Pay TV (M-net, DSTV, TOP TV)	
House/ cluster house/ town house		2 cell phones in household	
Electric stove		Home theatre system	
Tap water in house/ on plot		3 or more cell phones in household	
Microwave oven		Vacuum cleaner	
Flush toilet inside house		0 or 1 radio set in household	
Deep freezer – free standing		Motor vehicle in household	
Hot running water		Air conditioner (excludes fans)	
Washing machine		Computer- desktop, laptop	
Built in kitchen sink		1or more TVs	
No domestic workers or gardeners		Landline (exclude. cell phone)	
Swimming pool			

Section C: Belief statements

Fruit and vegetable intake

Eating fruits and vegetables every day will make me feel better physically.	Disagree—1—2—3—4—5—6—7-Agree
Eating fruits and vegetables every day will help control my weight.	Disagree—1—2—3—4—5—6—7-Agree
Eating less fruit will help control my blood sugar levels (i.e. to reduce the risk of diabetes).	Disagree—1—2—3—4—5—6—7-Agree
Vegetables do not take a long time to prepare	Disagree—1—2—3—4—5—6—7-Agree
Fruits and vegetables are affordable.	Disagree—1—2—3—4—5—6—7-Agree
Fruits and vegetables are easy to find in the stores/ shops nearby.	Disagree—1—2—3—4—5—6—7-Agree
I am confident that I can eat the recommended amount of fruits and vegetables every day.	Disagree—1—2—3—4—5—6—7-Agree
Most people who are important to me eat fruits and vegetables every day.	Disagree—1—2—3—4—5—6—7-Agree

Sugar	
Eating less sugary foods/snacks/ drinks will help reduce the risk of diseases e.g. diabetes.	Disagree—1—2—3—4—5—6—7-Agree
It is also important to limit my intake of sugary foods/snacks/drinks after the pregnancy.	Disagree—1—2—3—4—5—6—7-Agree
Decreasing the amount of sugary foods/snacks/ drinks I eat will help control my weight.	Disagree—1—2—3—4—5—6—7-Agree
Increasing the amount sugary foods/snacks/drinks I eat and drink make me feel unwell (tired, headache, dizzy, signs of hyper glycaemia etc).	Disagree—1—2—3—4—5—6—7-Agree
I want to reduce the amount of sugary foods/snacks/ drinks I eat and drink to prevent pregnancy/ birth complications.	Disagree—1—2—3—4—5—6—7-Agree
It is easy to exclude sugary foods/snacks/drinks from my daily diet.	Disagree—1—2—3—4—5—6—7-Agree
Foods/snacks/drinks that are low sugar/ sugar free are easy to find in my surroundings.	Disagree—1—2—3—4—5—6—7-Agree
Eating/drinking less sugary foods/snacks/drinks is up to me.	Disagree—1—2—3—4—5—6—7-Agree
Knowing how to control my cravings for sugary foods/snacks/ drinks during pregnancy will make it easier for me to eat less of these foods.	Disagree—1—2—3—4—5—6—7-Agree
Low sugar/ sugar-free foods/snacks/ drinks are expensive.	Disagree—1—2—3—4—5—6—7-Agree
Low sugar/ sugar-free foods taste good/ are tasty.	Disagree—1—2—3—4—5—6—7-Agree
People around me eat/serve sugary foods/snacks/drinks at most events/ functions (social, religious, or work events)	Disagree—1—2—3—4—5—6—7-Agree

Section D: Dietary intake assessment

NB! Think back to the last two weeks and divide the food cards into two piles i.e. foods you did eat and foods you did not eat.

A. Food Item (with FMP numbers)	*B. Description of food item*	*C. Amount consumed*	*D. Portion size*	*E. Times/day*	*F. Times/week*
**DAIRY – BLUE**					
1. Sugar in tea/coffee			Tbs/tsp heaped/level		
1. Sugar in cooking (veg/ porridge)			Tbs/tsp heaped/level		
2. Milk in tea/coffee	Full cream / low fat (2%)/ fat-free		Little / milky		
2.Milk with porridge	Full cream/ low fat (2%)/ fat-free				
3. Buttermilk/maas			Small or large glass		
4. Milk drinks			Small or large glass or ml		
5. Yoghurt	Plain / fruit & sweetened Fat: full / low / free		100ml tub/ 180ml tub/ heaped Tbs		
6. Cottage cheese	Fat: full / low / free		Heaped Tbs		
7. Hard Cheese	Type		Slice / matchbox		
8. Processed cheese	Type		Wedges/Tbs		
9. Ice cream & Ice lollies	Type		Scoops or heaped Tbs or nr of lollies		
**STARCH - BROWN**					
1. Brown bread/rolls			Slice		
1. White bread/rolls			Slice		
2. Whole wheat /Low GI bread			Slice		

A. Food Item (with FMP numbers)	*B. Description of food item*	*C. Amount consumed*	*D. Portion size*	*E. Times/day*	*F. Times/week*
2. Fat cakes			Small = 1 matchbox;<br>Med = 2 matchboxes<br>Lrg = 3 matchboxes		
3. Breakfast cereals	Specify type		½ or ¾ of a Bowl		
4. Maize porridge soft			Bowl		
4. Maize porridge stiff			Bowl		
4. Mabele/martabella soft			Bowl		
4. Mabele/ stiff			Bowl		
4. Oats			Bowl		
5. Pasta without sauce	White/ brown pasta		Heaped serving spoon		
6. Pasta dishes	White/ brown pasta		Heaped serving spoon		
7. Rice	White/brown		Heaped serving spoon<br>½ cup dough model		
7. Samp/mealie meal			Heaped serving spoon<br>½ cup dough model		
7. Wheat rice			Heaped serving spoon<br>½ cup dough model		
8. Pizza and savoury tart	Type		? pic in file		
**FATS - TAN**					
1. Brick margarine	Type		Tbs/tsp heaped/level		
1. Tub margarine	Type		Tbs/tsp heaped/level		
1. White margarine	Type		Tbs/tsp heaped/level		
1. Butter	Type		Tbs/tsp heaped/level		
2. Animal fat i.e lard					
3. Cream and substitutes			Tbs/tsp		
4. Oils	Sunflower / fish oil / canola oil / olive oil		Tbs/tsp		
5. Salad dressing			Tbs/tsp		
5. Mayonnaise			Tbs/tsp		
**SPREADS - PINK**					
Cheese spread	Type		Thin / med /thick		
Honey/syrup			Heaped Tbs/tsp		
Jam	Regular/low sugar		Heaped Tbs/tsp		
Peanut butter	Regular/no sugar		Heaped Tbs/tsp		
Sandwich spread	Type		Heaped Tbs/tsp		
**EGGS - YELLOW**					
Boiled			1 egg		
Fried			1 egg		
Omelette			1egg		

A. Food Item (with FMP numbers)	*B. Description of food item*	*C. Amount consumed*	*D. Portion size*	*E. Times/day*	*F. Times/week*
Scrambled			1 egg		
**FRUIT - ORANGE**					
1. Apples, pears			Small / med /large		
2. Bananas			Small / med /large		
6. Grapes			Nr of grapes		
8. Mango/paw paw					
9. Melons					
11. Oranges, Naartjies			Small / med /large		
12. Peaches			Small / med /large		
16. Dried fruit					
17. Fruit juice	Type		ml or small glass or tall glass		
**SOUP, LEGUMES, NUTS – pale green**					
1. Soups			Ladle/bowl		
2. Legumes & lentils			½ cup dough model		
3. Seeds & nuts, peanuts			Handful		
**FISH AND SEAFOOD - BEIGE**					
1. Fried fish			Per picture		
2. Grilled/smoked/dried fish	Type		Per picture		
3. Pilchards & sardines	In oil/brine/t. sauce		Tin		
3. Tuna - tinned	In oil/brine		Tin		
**MEAT - RED**					
1. Beef & Ostrich	Cut		Matchbox		
2. Patties & mince	Type: beef/ostrich Regular/lean/extra-lean		Small/medium		
3. Burgers & take-aways	Grilled/fried				
4. Chicken – with skin	Grilled/fried		Thigh / wing / drumstick / breast		
4. Chicken – without skin	Grilled/fried		Thigh / wing / drumstick / breast		
5. Cold meat	Type		slice		
7. Meat pies	Type		Size - ruler		
8. Mutton	Type		Line drawings		
9. Pork	Type		Line drawings		
10. Sausage & Vienna	Type		Ruler and thick or thin		
11. Traditional & organ meats	Type				
13. Dry sausage & biltong	Type				
**VEGETABLES - GREEN**					
Avocado			½ or ¼ etc.		
5. Orange/yellow veg (butternut,			½ cup dough model		

A. Food Item (with FMP numbers)	*B. Description of food item*	*C. Amount consumed*	*D. Portion size*	*E. Times/day*	*F. Times/week*
pumpkin, carrots, sweet potato, gem squash, mealies)					
6. Green veg (spinach, peas, green beans, broccoli)			½ cup dough model		
7. Cabbage, cauliflower, lettuce			½ cup dough model		
12. Mixed vegetables			½ cup dough model		
15. Potatoes			Nr med		
16. Potato chips			½ cup dough model		
20. Tomatoes			Nr or ½ cup		
**BISCUITS, CAKES, PUDDINGS**					
1. Biscuits/cookies	Type		nr		
2. Biscuits/savoury	Type		nr		
3. buns/muffins/scones	Type		Picture in file		
4. Cakes and tarts	Type		Line drawings		
5. Doughnuts/éclairs	Type		nr		
6. Pancakes/waffles	Type		nr		
7. Pudding/custard	Type		bowl		
8. Rusks			nr		
**SNACKS, SWEETS & COLD DRINKS - PINK**					
1. Carbonated cold drinks	Specify		ml or tin or small glass or tall glass		
1. Diet cold drinks	Specify		ml or tin or small glass or tall glass		
2. Energy drinks	Specify		ml or tin or small glass or tall glass		
2. Squashes	Specify		ml or tin or small glass or tall glass		
3. Crisps	Specify		small packet – 40g		
4. Sweets	Specify		nr		
4. Chocolates	Specify		50g bar or slab or nr of blocks from slab		
**SAUCES AND CONDIMENTS - GRAY**					
1. Cheese and white sauces	Specify		Tbs		
2. Tomato sauce & other	Specify		Tbs		
**ALCOHOLIC DRINKS - GRAY**					
1. Beer & cider & coolers			ml/bottles/shots		
2. Wine			ml/bottles/shots		
3. Spirits			ml/bottles/shots		
4. Liqueurs and fortified wine			ml/bottles/shots		
Other			ml/bottles/shots		

A. Food Item (with FMP numbers)	*B. Description of food item*	*C. Amount consumed*	*D. Portion size*	*E. Times/day*	*F. Times/week*

Section E: Physical activity (GPPAQ)	
**1. Please tell us the type and amount of physical activity involved in your work. Please tick one box only.**	
I am not employed (e.g. retired, retired for health reasons, unemployed, full-time carer etc.)	
I spend most of my time at work sitting (such as in an office)	
I spend most of my time at work standing or walking. However, my work did not require much intense physical effort (e.g. shop assistant, hairdresser, security guard, childminder, etc.)	
My work involves definite physical effort including handling of heavy objects and use of tools (e.g. plumber, electrician, carpenter, cleaner, hospital nurse, gardener, postal delivery workers etc.)	
My work involves vigorous physical activity including handling of very heavy objects (e.g. scaffolder, construction worker, refuse collector, etc.)	

2. During the last week, how many hours did you spend on each of the following activities?

	**None**	**Some but less than 1 hour**	**1 hour but less than 3 hours**	**3 hours or more**
Physical exercise such as swimming, jogging, aerobics, football, tennis, gym workout etc.				
Cycling, including cycling to work and during leisure time				
Walking, including walking to work, shopping, for pleasure etc.				
Housework/Childcare				
Gardening/DIY				
